# The impact of Oncotype DX breast cancer assay results on clinical practice: a UK experience

**DOI:** 10.1007/s10549-020-05578-6

**Published:** 2020-03-13

**Authors:** Valerie E. Crolley, Husam Marashi, Shabbir Rawther, Bhawna Sirohi, Marina Parton, Janine Graham, Anup Vinayan, Stephanie Sutherland, Anne Rigg, Anshu Wadhawan, Catherine Harper-Wynne, Emma Spurrell, Hannah Bond, Fharat Raja, Judy King

**Affiliations:** 1 Royal Free London NHS Foundation Trust, London, UK; 2 Beatson West of Scotland Cancer Centre, Glasgow, UK; 3 Sheffield Teaching Hospitals NHS Foundation Trust, Sheffield, UK; 4 Royal Marsden NHS Foundation Trust, London, UK; 5 South Tees NHS Foundation Trust, Middlesbrough, UK; 6 Luton & Dunstable NHS Trust, Luton, UK; 7 Mount Vernon Cancer Centre, Northwood, UK; 8 Guys and St Thomas NHS Foundation Trust, London, UK; 9 Velindre University NHS Trust, Cardiff, UK; 10 Maidstone and Tunbridge Wells NHS Trust, Maidstone, UK; 11 The Whittington Hospital NHS Trust, London, UK; 12 Royal Cornwall Hospitals NHS Trust, Truro, UK; 13 University College London Hospitals NHS Foundation Trust, London, UK; 14 North Middlesex University Hospital NHS Trust, London, UK; 15 Barts Health NHS Trust, London, UK

**Keywords:** Breast cancer, Adjuvant chemotherapy, Early breast cancer, Biomarkers, Oncotype DX breast recurrence score assay

## Abstract

**Background:**

Genomic tests are increasingly being used by clinicians when considering adjuvant chemotherapy for patients with oestrogen receptor-positive (ER+), human epidermal growth factor 2-negative (HER2−) breast cancer. The Oncotype DX breast recurrence score assay was the first test available in the UK National Health Service. This study looked at how UK clinicians were interpreting Recurrence Scores (RS) in everyday practice.

**Methods:**

RS, patient and tumour characteristics and adjuvant therapy details were retrospectively collected for 713 patients from 14 UK cancer centres. Risk by RS-pathology-clinical (RSPC) was calculated and compared to the low/intermediate/risk categories, both as originally defined (RS < 18, 18–30 and > 30) and also using redefined boundaries (RS < 11, 11–25 and > 25).

**Results:**

49.8%, 36.2% and 14% of patients were at low (RS < 18), intermediate (RS 18–30) and high (RS > 30) risk of recurrence, respectively. Overall 26.7% received adjuvant chemotherapy. 49.2% of those were RS > 30; 93.3% of patients were RS > 25. Concordance between RS and RSPC improved when intermediate risk was defined as RS 11–25.

**Conclusions:**

This real-world data demonstrate the value of genomic tests in reducing the use of adjuvant chemotherapy in breast cancer. Incorporating clinical characteristics or RSPC scores gives additional prognostic information which may also aid clinicians’ decision making.

**Electronic supplementary material:**

The online version of this article (10.1007/s10549-020-05578-6) contains supplementary material, which is available to authorised users.

## Introduction

Breast cancer is one of the most commonly diagnosed cancers in women worldwide, with over 250,000 new cases diagnosed in the US in 2017 (30% of the total new cancer diagnoses in women that year) [1]. The UK, like many developed countries, has a very high incidence of breast cancer, with 55,213 cases diagnosed in 2015, representing 31% of all female cancers [2]. However, while the incidence continues to rise, breast cancer-specific mortality has fallen sharply—in part due to earlier detection, and in part due to improved adjuvant therapies [3].

The current UK standard of care in early-stage (node-negative) oestrogen receptor-positive (ER+), human epidermal growth factor 2-negative (HER2−) breast cancer is surgery followed by adjuvant endocrine therapy, with or without the addition of systemic chemotherapy [4, 5]. However, not all patients require adjuvant chemotherapy and endocrine therapy after surgery. Given the short- and long-term toxicity associated with adjuvant chemotherapy [6–8], patient identification and selection is crucial—for both the patients at high risk of recurrence who are most likely to benefit from chemotherapy, as well as for patients at low risk of recurrence in whom chemotherapy may safely be avoided.

However, there is also a group of patients at intermediate risk of recurrence, for whom the benefit of adjuvant chemotherapy remains unclear. For these patients, gene expression assays are increasingly used to aid clinical decision making. In 2013, the UK National Institute for Health and Care Excellence (NICE) recommended that the Oncotype DX breast recurrence score assay (Genomic Health, Redwood City, CA) was made available for patients at intermediate risk of recurrence with node-negative, ER+HER2− cancers, where the test result would help guide clinical decisions regarding adjuvant chemotherapy [4] and Oncotype DX testing was endorsed by the Molecular Pathology Evaluation Panel (MPEP) in NHS Scotland in March 2016.

The Oncotype DX breast recurrence score assay is a 21 gene panel developed to predict the risk of tumour recurrence in patients with ER+HER2− breast cancer [9]. The expression of 16 genes is used to calculate a recurrence score (RS) on a scale of 0–100 [9]. The calculated scores were then grouped into three risk categories: low risk (RS ˂ 18), intermediate risk (RS 18–30) and high risk (RS ≥ 31) [10].

Prior to the publication of the results of the Trial Assigning Individualized Options for Treatment (TAILORx) clinical trial in patients with intermediate RS, there were limited data to guide clinicians in the management of this patient group. This trial incorporated modified definitions of risk when compared to those defined in the development of Oncotype DX, with low, intermediate and high risk defined as RS of < 11, 11–25 and > 25, respectively [11, 12]. In TAILORx, patients with intermediate RS (11–25) were randomised to endocrine therapy versus chemotherapy plus endocrine therapy [12]. The results demonstrated no significant difference between the two arms for invasive disease-free survival (83.3% vs. 84.3% in the endocrine and chemo-endocrine therapy groups, respectively), disease recurrence and overall survival (93.9% vs. 93.8%) [11]. Although chemotherapy was found to be of some benefit in women aged under 51 years with a RS of 16–25, this has been hypothesised to be due to the effect of chemotherapy induced amenorrhoea [11, 13].

Unlike other gene expression arrays, Oncotype DX does not incorporate other clinical or pathological risk factors which are known to be predictors of prognosis, such as tumour size and nodal status. However, RS scores from Oncotype DX can be combined with tumour grade, size and patient age standardised against the type of endocrine treatment used (either an aromatase inhibitor or tamoxifen) to create an integrated risk estimate [14, 15]. This produces a combined RS/pathological/clinical (RSPC) risk score, with risk classified as low (< 12%), intermediate (12–20%) or high (> 20%) [14]. Some oncologists utilise the RSPC score alongside the RS score to inform their decision on whether to recommend chemotherapy. Tang et al. originally showed that RSPC classified more patients as low risk than RS [14]. However, this is in contrast to data from a contemporary UK patient population [15], which is likely to be due to differences in the clinical characteristics of the patient populations being tested.

The aims of this study were to:

- Review real-world data to see how UK oncologists were interpreting the results of Oncotype DX tests in clinical practice, prior to the publication of results from the TAILORx trial,
- See how the modified definitions of RS and risk in TAILORx would affect the UK real-world clinical population and UK practice, and
- Review the correlation between RS and RSPC scores, and to see whether the correlation between risk categories improved following the adoption of the modified TAILORx definitions of RS and high risk.

## Methods

### Data sources and study population

Anonymised data were collected from a total of 14 cancer centres across the UK (Online Appendix 1), from patients who had undergone Oncotype DX testing between December 2015 and February 2018 as part of their routine clinical care, as per the 2013 NICE guidance for early-stage breast cancer [4].

All patients included in this study had been newly diagnosed with ER+HER2−, node-negative breast cancer, had undergone surgery with curative intent, were intended to receive endocrine therapy and were being considered for adjuvant chemotherapy. Patients with micro-metastases (≤ 2 mm axillary node metastasis) were included, but patients with nodal macro-metastases were excluded. All patients were at intermediate risk of disease recurrence, defined as either a Nottingham Prognostic Index score of > 3.4 [16] or a 3–5% increase in 10-year survival with 2nd generation chemotherapy according to NHS predict [4, 17].

Data on both patient and tumour characteristics were collected retrospectively from electronic and paper records. Data collected on patient characteristics included age, menopausal status and gender, while tumour characteristics collected included tumour size, grade, ER/PR/HER2 and nodal status. The Oncotype DX recurrence score was recorded, as well as information on whether or not the patient was subsequently offered chemotherapy, which regimens were offered and whether or not the patient received chemotherapy. Whether or not patients were offered or recommended to have chemotherapy was coded from electronic patient records and patient letters at the investigator’s discretion. Data were initially analysed in line with the published risk categories of < 18, 18–30 and > 30 for low, intermediate and high risk, respectively, based on the NSAPB B-20 trial [9]. Data were also re-analysed using the amended risk categories adopted in the TAILORx clinical trial: < 11, 11–25 and > 25 for low, intermediate and high risk, respectively [12].

RS% and RSPC analysis was performed using the web based tool on the genomic health website/physician portal [18]. RS was combined with tumour size, grade, patient age and intended endocrine therapy to calculate RSPC. Where data were unavailable on the intended endocrine therapy, tamoxifen was the assumed endocrine therapy in women aged < 51 years, and an aromatase inhibitor for patients aged 51 and above, in line with 51 being the average age of the menopause in the UK [19]. Previously published boundaries of < 12%, 12–20% and > 20% risk of distant recurrence at 10 years were used to categorise low, intermediate and high risk by RSPC [14]. Although a 12% risk of distant metastases can be considered to be relatively high, we have chosen to use the generally accepted boundaries for RSPC in order to aid comparison with results published by other groups.

### Statistical analysis

The percentage of patients either being offered or receiving chemotherapy was calculated according to RS, tumour grade, tumour size and patient age. Cases with missing data were excluded from analysis, and cases with missing data are noted in the results. Chi-squared tests were performed to determine the significance of relationships and *p* ≤ 0.05 was seen to be significant. Data and statistical analysis was carried out using Microsoft Excel (Microsoft Corporation, Redmond VA), and graphs were created using ggplot2 and reshape2 in R [20, 21].

## Results

### Patient and tumour characteristics

Data were collected from a total of 713 patients. Information was collected from 14 NHS trusts from across the UK (Supplementary Table 1), with a median of 38 patients from each site (range 5 to 136). Baseline patient and tumour characteristics are shown in Table 1. The median age was 55 (range 28–80) and the median tumour size was 24 mm (range 4.5–106 mm).

**Table 1:** Baseline patient and tumour characteristics

Total					
713					

Age (%)					
≤ 39	40–49	50–59	60–69	≥ 70	
5.0	22.3	36.2	27.8	8.7	

Gender (%)					
Female			Male		
100			0		

Tumour grade					
Grade 1	Grade 2		Grade 3		Data not available
10	351		307		45

Tumour size					
< 20 mm	20–29 mm	30–39 mm	40–49 mm	≥ 50 mm	Data not available
205	276	121	60	49	2

Nodal status					
Node negative	Micro-metastases			Data not available	
641	27			45	

### Recurrence score (RS) and whether chemotherapy was recommended

**Fig. 1:**
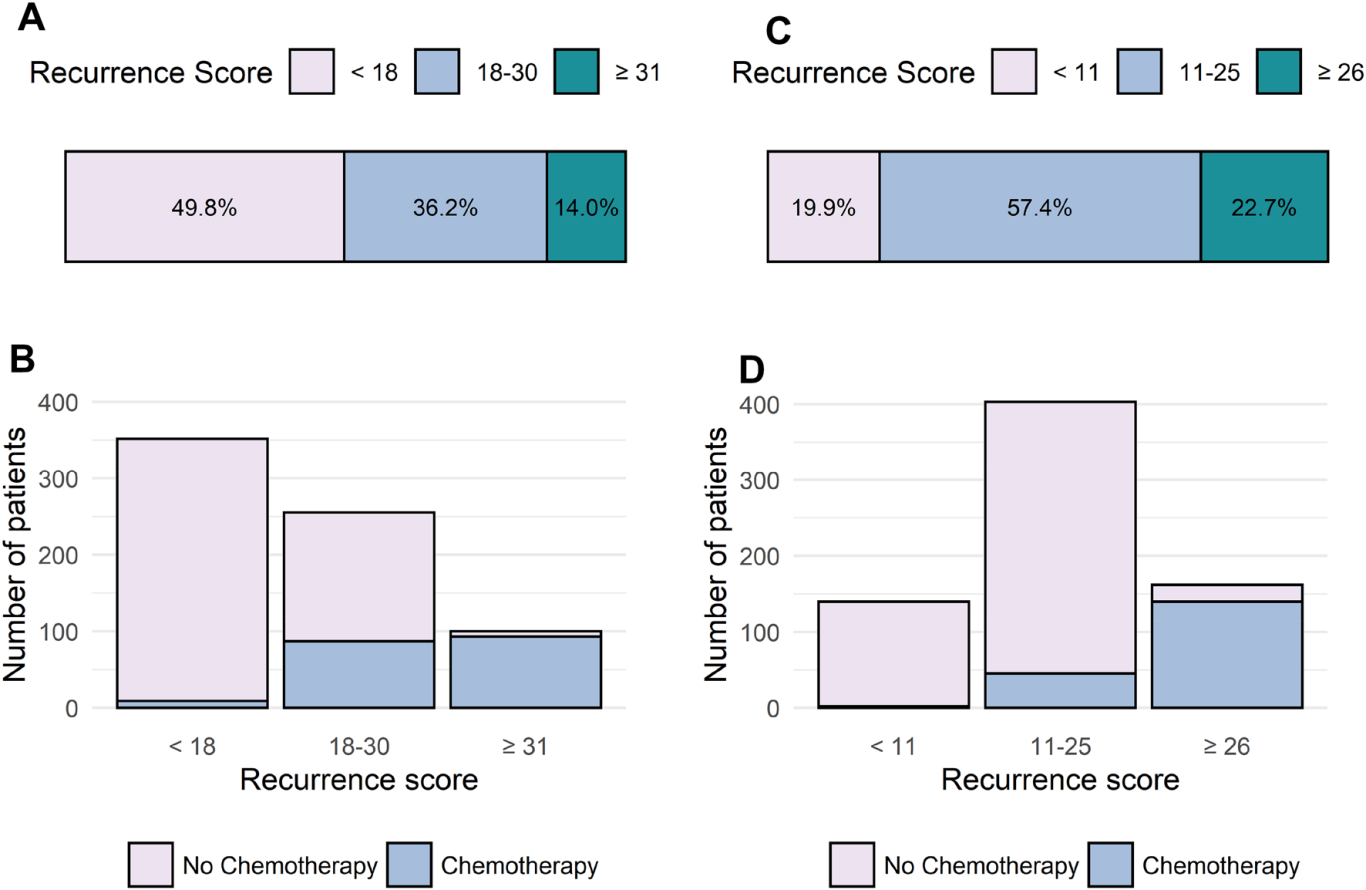
**a** Distribution of patients by recurrence score (RS); **b** chemotherapy use according to RS; **c**, **d** distribution of patients and chemotherapy use according to the revised TAILORx definitions of low-, intermediate- and high-risk RS

355 (49.8%) patients were in the low-risk category (recurrence score < 18), 258 (36.2%) were intermediate risk (score 18–30) and 100 (14.0%) were high risk (score ≥ 31) (Fig. 1a). The mean recurrence score was 19, with a range 0 to 76.

**Fig. 2:**
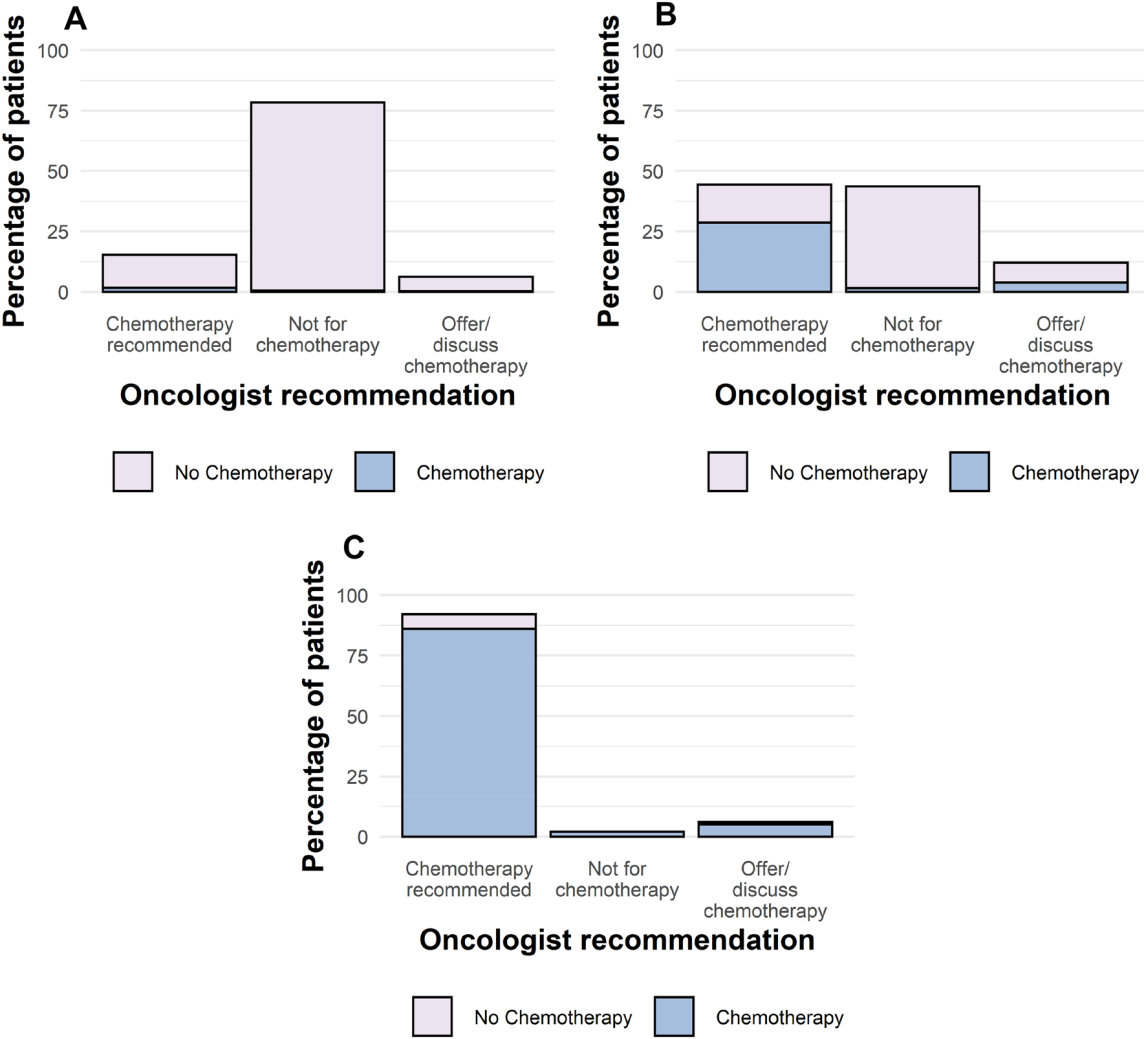
Oncologists’ recommendation to patients, and subsequent uptake of chemotherapy in patients with **a** low-risk recurrence score (RS) (< 18); **b** intermediate-risk RS (18–30); **c** high-risk RS (> 30)

Chemotherapy was recommended for 54/352 (15.3%) patients with a low RS (< 18) (3 patients were excluded from analysis due to incomplete data), and was discussed or offered to a further 22 (Fig. 2a). Of note, only 5 of these patients (4 recommended and 1 offered) had T3 tumours (tumour size > 50 mm). Higher grade tumours however were more likely to be recommended or offered chemotherapy than patients with lower grade tumours; 32.5% (36/111) of patients with Grade 3 tumours were recommended or offered chemotherapy compared to 11.9% (25/210) of patients with Grade 2 tumours. Chemotherapy was recommended for 92/100 (92.0%) of high-risk patients (Fig. 2c). For patients with intermediate RS (18–30), chemotherapy was recommended for 113/255 patients (44.3%) (3 patients were excluded from analysis due to incomplete data), the option of chemotherapy was discussed or offered to 31 (12.2%), while 111 (43.5%) were not offered chemotherapy (Fig. 2b).

63.7% (165/259) of patients went on to receive chemotherapy when oncologists *recommended* chemotherapy, while 27.1% (*n* = 16/59) of patients went on to receive chemotherapy when it had been *offered or discussed* as an option. In patients for whom complete data were available, 189/707 patients (26.7%) received chemotherapy, nearly half of whom (49.2%, 93/189) had RS > 30, while 46.0% (87/189) patients had RS 18–30 and 4.8% (9/189) had RS < 18 (Fig. 1b). 93.3% of patients who received chemotherapy had RS > 25.

When patients went on to receive chemotherapy, an anthracycline only regime was most common, accounting for 72.8% of cases, 29.9% FEC75 (5-fluorouracil, epirubicin [75 mg/m^2^] and cyclophosphamide), 21.7% EC (epirubicin and cyclophosphamide), 10.9% FEC80 (5-fluorouracil, epirubicin [80 mg/m^2^] and cyclophosphamide) and 10.3% AC (doxorubicin and cyclophosphamide). 10.3% of patients received a 3rd generation chemotherapy regimen containing both an anthracycline and a taxane [22] [this included FEC-T (5-fluorouracil, epirubicin, cyclophosphamide and docetaxel), AC-T (doxorubicin, cyclophosphamide and docetaxel) and EC-T (epirubicin, cyclophosphamide and paclitaxel)].

### Intermediate recurrence score (18–30) subgroup analysis

Patients with RS in the higher end of the intermediate range were more likely to be offered chemotherapy: 87.1% (54/62) versus 46.4% (89/192) for RS 26–30 versus RS 18–25, respectively (data were not available for 1 patient) (*p* < 0.01). Oncologists recommended chemotherapy more frequently for higher grade tumours with intermediate RS: 61.0% (72/118) of patients with Grade 3 and 30.6% (38/124) with Grade 2 cancers, and there was a weak correlation between tumour grade and recurrence score (*R* = 0.329) (information on tumour grade was not available for 11 patients with intermediate-risk RS) (*p* < 0.01). The size of the tumour was less well correlated with whether patients with intermediate RS were offered chemotherapy, as was the age of the patient, and RS was not correlated with either the age of the patient (*R* = 0.047) or the size of the tumour (*R* = − 0.104) (*p* < 0.01).

### Reclassification of real-world data using revised low- and intermediate-RS cutoffs

The TAILORx trial reclassified risk as RS < 11 (low risk), 11–25 (intermediate) and > 25 (high risk). Using this classification, the high-risk cutoff is reduced from 30 to 25. When this classification is applied to our dataset, the proportion of patients who were low, intermediate and high risk was 19.9%, 57.4% and 22.7%, respectively (Fig. 1c). Re-analysing the data with the reclassified RS score used in TAILORx resulted in a decrease in the proportion of "intermediate-risk" patients who received chemotherapy (Fig. 1d).

### RSPC scores

RSPC scores were calculated for 665 patients for whom complete data were available. RSPC scores and risk categories were then compared to the RS scores, firstly using the original RS risk categories and secondly with the amended RS risk categories used in the TAILORx trial. The results are shown in Table 2.

**Table 2:** Comparison of the risk assigned by RSPC compared to (1) the original Oncotype DX recurrence score definitions of low, intermediate and high risk and (2) the low-, intermediate- and high-risk recurrence score definitions used in the TAILORx clinical trial

		Risk assigned by RS		
		Original definition		
		Low (< 18)	Intermediate (18–30)	High (≥ 31)
Risk assigned by RSPC	Low (< 12%)	176 (54.0%)	37 (15.2%)	1 (1.1%)
	Intermediate (12—20%)	117 (35.9%)	119 (48.8%)	16 (16.8%)
	High (> 20%)	33 (10.1%)	88 (36.1%)	78 (82.1%)
	Total	326 (100%)	244 (100%)	95 (100%)

		TAILORx		
		Low (< 11)	Intermediate (11–25)	High (≥ 26)
Risk assigned by RSPC	Low (< 12%)	94 (72.9%)	114 (29.7%)	6 (3.9%)
	Intermediate (12–20%)	30 (23.3%)	181 (47.1%)	41 (27.0%)
	High (> 20%)	5 (3.9%)	89 (23.2%)	105 (69.1%)
	Total	129 (100%)	384 (100%)	152 (100%)

Concordance between RS and RSPC scores was variable. For patients with low RS (RS < 18), 54.0% were also classified as low risk by RSPC (Table 2). However, 35.9% and 10.1% were reclassified as intermediate and high risk by RSPC, respectively. For patients with intermediate RS (RS 18–30), 48.8% were also classified as intermediate risk by RSPC. 15.2% were downgraded to low risk and 36.1% were upgraded to high risk by RSPC. For patients with high-risk RS (RS > 30), 82.1% were also classified as high risk by RSPC. 16.8% and 1.1% were reclassified as intermediate and low risk, respectively.

Applying the revised RS boundary scores from the TAILORx study improved the concordance between RS and RSPC for patients in the low-risk group (RS < 11), where concordance with low-risk RSPC improved to 72.9% (Table 2). Fewer patients were reclassified as intermediate risk (23.3%) and 3.9% of patients were reclassified as high risk. For intermediate-risk RS (11–25), concordance with intermediate RSPC was similar at 47.1%. However, fewer patients were reclassified as high risk: 23.2% vs 36.1% previously; a higher proportion of patients were reclassified as low risk by RSPC: 29.7% vs 15.2% previously. Concordance between high-risk RS and RSPC decreased to 69.1% (previously 82.1%), with a higher proportion of patients being reclassified as intermediate risk according to RSPC.

## Discussion

These results from a real-world series of over 700 UK patients show that 73.3% of those who underwent the Oncotype DX assay did not receive adjuvant chemotherapy. 49.8% of patients had a low-risk RS (< 18), which is in keeping with other UK results [23], and is similar to the results found in a meta-analysis of international studies [24].

Interpretation of RS scores may vary between clinicians or institutions, which may in part reflect varying levels of familiarity with the test; in our data the range of the number of tests performed per institution ranged from 5 to 136. Our data demonstrate the greatest uniformity of clinical practice in patients with high or low RS: 93.0% of patients with a high RS (> 30) received chemotherapy, compared with only 2.6% of patients with a low RS (< 18) which is in keeping with a retrospective review of the NSABP-20 trial [9]. NSABP-20 also demonstrated that patients with a low RS (< 18) derived no significant benefit from chemotherapy [9] and the data from the low RS (< 11) arm of the TAILORx clinical trial showed that endocrine therapy alone is associated with good long-term outcomes, which indicates that patients with low RS should generally avoid chemotherapy [25, 26]. When patients were offered chemotherapy, there was a wide variation in the chemotherapy regimens prescribed. The majority received an anthracycline only regime (71.7% receiving FEC, EC or AC) with 10.3% receiving a 3rd generation combination of anthracycline and a taxane. There was no correlation between recurrence score and the type of chemotherapy prescribed (data not shown) and decisions on chemotherapy regime appeared to be institution dependent.

The greatest variation in clinical practice was seen in patients with intermediate risk scores, which likely reflects the lack of clinical data during the period of data collection (prior to publication of the TAILORx trial). There was also a lack of clear consensus in the ESMO, ASCO and NCCN clinical guidelines, which at the time of writing do not specify which RS scores justify chemotherapy [5, 27–29]. In the randomised phase 3 TAILORx trial, over 6000 patients with (reclassified) intermediate RS 11–25 were randomly assigned to either chemotherapy plus endocrine therapy or endocrine therapy alone [11]. The results from the intermediate-risk group showed no difference in disease-free survival or overall survival between the two arms, and the authors concluded that patients with RS < 26 can be spared chemotherapy [11] (Patients with RS < 11 receiving endocrine therapy alone had previously been shown in the TAILORx trial to have a very low risk of disease recurrence [12]).

However, it is important to note that the patients included in the TAILORx study were a low-risk population, with smaller tumours and lower tumour grades than the average UK population: < 15% of tumours in TAILORx were grade 3, compared with almost 50% in this UK series; the vast majority of tumours enrolled in TAILORx were < 2 cm, whereas 71% of tumours in our series were T2 or T3. Hence, approximately 80% of the patients in TAILORx would be deemed too low risk to qualify for Oncotype DX testing in the UK. This needs to be borne in mind when applying the trial data to the real-world population, who may have higher risk clinical characteristics and for whom the RSPC score may provide useful additional prognostic information.

In this series, 46% of patients classed as low risk by Oncotype DX (RS < 18) were classified as intermediate or high risk when tested with RSPC. Incorporating the revised TAILORx RS cutoffs of < 11, 11–25 and > 25 improved concordance between RS and RSPC to over 70% for the low- and high-RS groups. However, in our study > 50% of patients with intermediate RS (11–25) were reclassified as either high or low risk by RSPC. The clinical characteristics of the patients in our series differed from that of the majority of patients enrolled in TAILORx: patients in our series were more likely to have tumours > 2 cm in size and/or be Grade 3. This is likely to account for the discrepancies between risks as defined by RS vs RSPC in our patient population. Dodson et al*.* also noted that RSPC should potentially be taken into account when deciding if a patient should have adjuvant endocrine therapy alone [15]. However, it should be noted that while high RSPC scores may indicate a higher risk of recurrence, it is not clear whether patients with high-risk RSPC would benefit from chemotherapy as this hypothesis has not been tested in a clinical study.

Ongoing trials will further evaluate the role genomic tests will play in helping clinicians determine whether patients with higher risk clinical characteristics should be offered chemotherapy. The RxPONDER trial randomised node-positive/ER-positive/HER2-negative patients with RS < 25 to receive either chemotherapy plus endocrine therapy or endocrine therapy alone [30], and results are awaited. The Optimal Personalised Treatment of early breast cancer usIng Multiparameter Analysis (OPTIMA) clinical trial is currently ongoing in the UK, and randomises high-risk patients to either a Prosigna test or to the current standard of care (chemotherapy). Patients with a high-risk Prosigna test will receive chemotherapy, and those whose test results show them to be low risk will receive endocrine therapy alone [31, 32]. These and further upcoming clinical trials will further guide clinicians in determining which patients are most likely to benefit chemotherapy, and in which patients chemotherapy may be safely avoided.

## Electronic supplementary material

Below is the link to the electronic supplementary material.

Supplementary file1 (DOC 50 kb)

## Data Availability

All data were collected from NHS clinical records. The datasets generated and/or analysed during the current study are available from the corresponding author on reasonable request.

## References

[1] Siegel RL, Miller KD, Jemal A (2017). Cancer statistics, 2017. CA Cancer J Clin.

[2] Cancer Research UK (2018) Breast cancer incidence (invasive) statistics. In: Cancer Res. UK. https://www.cancerresearchuk.org/health-professional/cancer-statistics/statistics-by-cancer-type/breast-cancer/incidence-invasive. Accessed 4 Jun 2018

[3] Munoz D, Near AM, Van Ravesteyn NT (2014). Effects of screening and systemic adjuvant therapy on ER-specific US breast cancer mortality. J Natl Cancer Inst.

[4] National Institute for Health and Care Excellence (2013). Gene expression profiling and expanded immunohistochemistry tests for guiding adjuvant chemotherapy decisions in early breast cancer management : MammaPrint, Oncotype DX, IHC4 and Mammostrat. NICE Diagn Guid.

[5] National Cancer Institute (2017). NCCN guidelines: breast cancer. Natl Compr Cancer Netw Version.

[6] Matyszewski A, Czarnecka AM, Stachowiak P (2017). Cardiac safety of systemic therapy in breast cancer patients with high risk of atherosclerosis complications. Future Oncol.

[7] Tao JJ, Visvanathan K, Wolff AC (2015). Long term side effects of adjuvant chemotherapy in patients with early breast cancer. Breast.

[8] Peto R, Davies C, Early Breast Cancer Trialists’ Collaborative Group (EBCTCG) (2012). Comparisons between different polychemotherapy regimens for early breast cancer: meta-analyses of long-term outcome among 100,000 women in 123 randomised trials. Lancet (London, England).

[9] Paik S, Shak S, Tang G (2004). A multigene assay to predict recurrence of tamoxifen-treated, node-negative breast cancer. N Engl J Med.

[10] Sparano JA, Paik S (2008). Development of the 21-gene assay and its application in clinical practice and clinical trials. J Clin Oncol.

[11] Sparano JA, Gray RJ, Makower DF (2018). Adjuvant chemotherapy guided by a 21-gene expression assay in breast cancer. N Engl J Med.

[12] Sparano JA, Gray RJ, Makower DF (2015). Prospective validation of a 21-gene expression assay in breast cancer. N Engl J Med.

[13] Sparano JA, Gray R (2019). TAILORx: questions answered, lessons learned, and remaining knowledge gaps. J Clin Oncol.

[14] Tang G, Cuzick J, Costantino JP (2011). Risk of recurrence and chemotherapy benefit for patients with node-negative, estrogen receptor-positive breast cancer: recurrence score alone and integrated with pathologic and clinical factors. J Clin Oncol.

[15] Dodson A, Okonji D, Assersohn L (2018). Discordance between oncotype DX recurrence score and RSPC for predicting residual risk of recurrence in ER-positive breast cancer. Breast Cancer Res Treat.

[16] Todd JH, Dowle C, Williams MR (1987). Confirmation of a prognostic index in primary breast cancer. Br J Cancer.

[17] Wishart GC, Azzato EM, Greenberg DC (2010). PREDICT: a new UK prognostic model that predicts survival following surgery for invasive breast cancer. Breast Cancer Res.

[18] Genomic Health (2019) Genomic Health Physician Portal. www.genomichealth.com. Accessed 19 May 2019

[19] Mishra G, Hardy R, Kuh D (2007). Are the effects of risk factors for timing of menopause modified by age? Results from a British birth cohort study. Menopause.

[20] Wickham H (2016). ggplot2: elegant graphics for data analysis.

[21] Wickham H (2007). Reshaping data with the reshape package. J Stat Softw.

[22] Anampa J, Makower D, Sparano JA (2015). Progress in adjuvant chemotherapy for breast cancer: an overview. BMC Med.

[23] Loncaster J, Armstrong A, Howell S (2017). Impact of Oncotype DX breast Recurrence Score testing on adjuvant chemotherapy use in early breast cancer: real world experience in Greater Manchester, UK. Eur J Surg Oncol.

[24] Carlson JJ, Roth JA (2013). The impact of the Oncotype Dx breast cancer assay in clinical practice: a systematic review and meta-analysis. Breast Cancer Res Treat.

[25] Paik S, Tang G, Shak S (2006). Gene expression and benefit of chemotherapy in women with node-negative, estrogen receptor-positive breast cancer. J Clin Oncol.

[26] Sparano JA, Gray RJ, Makower DF (2015). Prospective validation of a 21-gene expression assay in breast cancer. N Engl J Med.

[27] Senkus E, Kyriakides S, Ohno S (2015). Primary breast cancer: ESMO Clinical Practice Guidelines for diagnosis, treatment and follow-up. Ann Oncol.

[28] Harris LN, Ismaila N, McShane LM (2016). Use of biomarkers to guide decisions on adjuvant systemic therapy for women with early-stage invasive breast cancer: American Society of Clinical Oncology clinical practice guideline. J Clin Oncol.

[29] Krop I, Ismaila N, Andre F (2017). Use of biomarkers to guide decisions on adjuvant systemic therapy for women with early-stage invasive breast cancer: American Society of Clinical Oncology clinical practice guideline focused update. J Clin Oncol.

[30] Jasem J, Fisher CM, Amini A (2017). The 21-gene recurrence score assay for node-positive, early-stage breast cancer and impact of RxPONDER Trial on chemotherapy decision-making: have clinicians already decided?. J Natl Compr Canc Netw.

[31] Bartlett J, Canney P, Campbell A (2013). Selecting breast cancer patients for chemotherapy: the opening of the UK OPTIMA Trial. Clin Oncol.

[32] Stein RC, Dunn JA, Bartlett JMS (2016). OPTIMA prelim: a randomised feasibility study of personalised care in the treatment of women with early breast cancer. Health Technol Assess (Rockv).

