# Supplementary material for: The impact of Oncotype DX breast cancer assay results on clinical practice: a UK experience
Source: Breast Cancer Res Treat. 2020 Mar 13;180(3):809–17. doi: 10.1007/s10549-020-05578-6 (PMC7103011; doi:10.1007/s10549-020-05578-6)
Supplement: Supplementary file 1 — Supplementary file1 (DOC 50 kb) [file 10549_2020_5578_MOESM1_ESM.doc]

Supplementary Table 1: The UK cancer centres which provided patient data.

| **List of institutions** |
| --- |
| Barts Health NHS Trust |
| Beatson West of Scotland Cancer Centre |
| Guy's and St Thomas' NHS Foundation Trust |
| The Hillingdon Hospitals NHS Foundation Trust |
| Luton & Dunstable NHS Trust |
| Maidstone and Tunbridge Wells NHS Trust |
| Royal Cornwall Hospitals NHS Trust |
| Royal Free London NHS Foundation Trust |
| The Royal Marsden NHS Foundation Trust |
| Sheffield Teaching Hospitals NHS Foundation Trust |
| South Tees Hospitals NHS Foundation Trust |
| University College London Hospitals NHS Foundation Trust |
| Velindre NHS Trust |
| Whittington Health NHS Trust |
